# High-definition tDCS of the temporo-parietal cortex enhances access to newly learned words

**DOI:** 10.1038/s41598-017-17279-0

**Published:** 2017-12-05

**Authors:** Garon Perceval, Andrew K. Martin, David A. Copland, Matti Laine, Marcus Meinzer

**Affiliations:** 1 The University of Queensland, Centre for Clinical Research, Brisbane, Australia; 2 The University of Queensland, School of Rehabilitation Sciences, Brisbane, Australia; 3 Åbo Akademi University, Department of Psychology, Turku, Finland

## Abstract

Learning associations between words and their referents is crucial for language learning in the developing and adult brain and for language re-learning after neurological injury. Non-invasive transcranial direct current stimulation (tDCS) to the posterior temporo-parietal cortex has been suggested to enhance this process. However, previous studies employed standard tDCS set-ups that induce diffuse current flow in the brain, preventing the attribution of stimulation effects to the target region. This study employed high-definition tDCS (HD-tDCS) that allowed the current flow to be constrained to the temporo-parietal cortex, to clarify its role in novel word learning. In a sham-controlled, double-blind, between-subjects design, 50 healthy adults learned associations between legal non-words and unfamiliar object pictures. Participants were stratified by baseline learning ability on a short version of the learning paradigm and pairwise randomized to active (20 mins; N = 25) or sham (40 seconds; N = 25) HD-tDCS. Accuracy was comparable during the baseline and experimental phases in both HD-tDCS conditions. However, active HD-tDCS resulted in faster retrieval of correct word-picture pairs. Our findings corroborate the critical role of the temporo-parietal cortex in novel word learning, which has implications for current theories of language acquisition.

## Introduction

Forming associations between words and their referents is an essential component of language acquisition across the lifespan^1^. Enhancing the effectiveness of this process would not only benefit the growing number of people required to learn a second language due to increasing transnational mobility worldwide, but also those who show impairments in this process due to developmental^2,3^ or acquired disorders such as post-stroke aphasia^4^.

Recent studies have suggested that transcranial direct current stimulation (tDCS), which uses a weak electrical current to modulate excitability of targeted brain regions^5^, can improve language functions like verbal fluency, picture naming, grammatical decisions and word list learning in health and disease (for reviews^6,7^). However, to date, few studies have investigated potential beneficial effects of tDCS on novel word learning, which serves as an experimental proxy for verbal associative learning ability^8^. Such paradigms require participants to establish new associations between objects (either familiar or unfamiliar) and novel word forms (i.e., legal non-words), a process that is supported by a widespread network of brain regions including the medial temporal lobe and fronto-temporal and parietal cortices^1,8,9^. Except for studies that investigated action word learning and stimulated the primary motor cortex^10,11^, all previous tDCS studies employing novel word learning paradigms in healthy young and older individuals targeted the posterior temporo-parietal cortex (CP5 of the EEG 10–20 system)^12–16^. This region was chosen here as well because of its importance in word-form processing and phonological working memory^1,17^. Despite differences regarding the experimental paradigms (e.g., explicit vs. implicit learning, real vs. abstract vs. blurred objects), mode of retrieval (e.g., spontaneous naming vs. receptive matching tasks) or stimulation parameters (e.g., intensity, duration, timing of tDCS), excitatory “anodal” tDCS of this region in the left hemisphere improved learning success for novel picture-word association^12–15^. One recent study though, reported improvement only in healthy elderly but not young participants^16^.

Importantly, all previous studies employed the so-called “conventional tDCS set-up” where the current was projected between two electrodes attached over the target region (anode: left CP5) and the right fronto-polar cortex (reference electrode). Such set-ups do not allow for the attribution of stimulation effects to the target region, because the current may affect regions in between the two electrodes^18,19^, including parts of the domain general “multi-demand cortex”, which has been shown to be important for novel word learning^9^. Moreover, except for one study that used a larger fronto-polar reference electrode^14^, which renders stimulation of this region inefficient^20^, the use of a small reference electrode in the remaining studies may have resulted in inhibition of the right prefrontal cortex. Therefore, the locus of the stimulation effect in all previous studies remains unclear.

To this end, the present study investigated for the first time whether focal current delivery by “high-definition” (HD) tDCS to the temporo-parietal cortex can improve novel word learning. We employed an established set-up that uses two concentric rubber electrodes (i.e., a small centre electrode attached over CP5 and a surrounding ring electrode that constrains the current flow to the target region^21,22^). This set-up has recently been shown to result in regionally specific^23^ or task specific^24^ behavioral modulation in other cognitive domains (i.e., inhibitory control, social cognition). Because the set-up is also compatible with functional magnetic resonance imaging (fMRI)^22^, the secondary goal of this study was to design a paradigm that can easily be implemented in an fMRI environment, to investigate the neural mechanisms underlying potential beneficial HD-tDCS effects in future studies. Our working hypothesis was that excitatory HD-tDCS of the temporo-parietal cortex would improve novel word learning compared to placebo HD-tDCS of the same region.

## Methods

### Study overview

**Figure 1:**
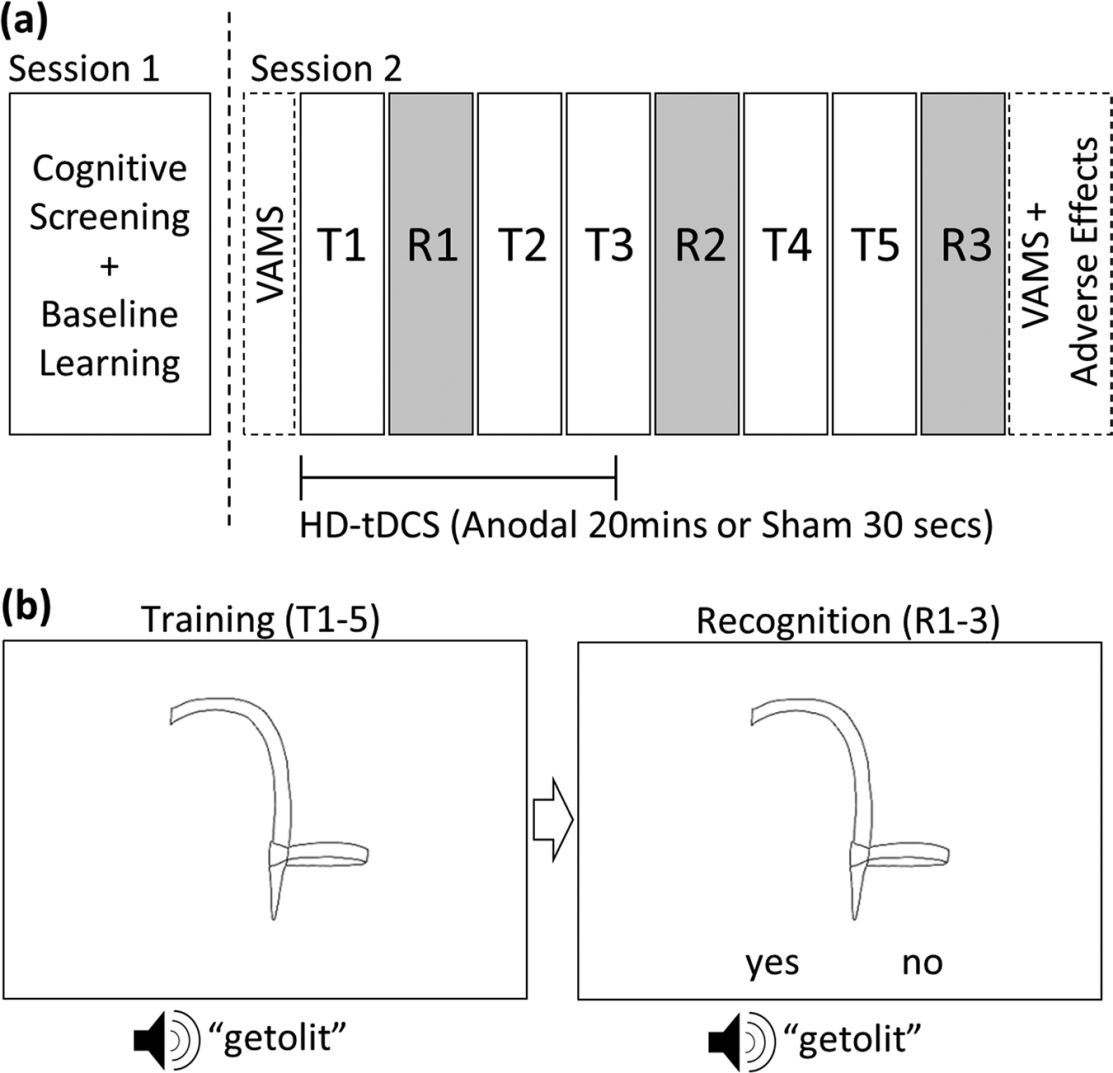
Study design and learning paradigm. **(a)** Illustrates the study design. At session 1, subjects completed a cognitive screening battery and the short version of the learning paradigm. At session 2, subjects received sham or anodal HD-tDCS while completing the associative learning paradigm. Three recognition blocks (R1–3) were interspersed throughout five training blocks (T1–5). Mood (Visual Analogue Mood Scale, VAMS) was assessed before and after training. Adverse effects were assessed at the end of training. **(b)** Illustrates novel object - name pairings presented during the training (T1–5) and recognition (R1–3) blocks. A novel object picture was displayed with a simultaneous auditory presentation of a non-word. At the recognition blocks, subjects were instructed to indicate whether the pairing shown was correct via a yes/no response using the computer keyboard.

The study employed a double-blind, sham tDCS controlled, between-subjects design and was conducted at the Centre for Clinical Research of the University of Queensland, Australia. Fifty healthy, right-handed adults participated in this study. We employed an explicit associative learning paradigm wherein participants were trained to learn associations between pictures of novel “ancient Finnish farming equipment” objects^8^ and non-words. The training was delivered during a single experimental session with concurrent active (anodal) or placebo (sham) HD-tDCS of the left posterior temporal lobe (PTL; N = 25 per group). Prior to the experiment, all participants were screened for baseline cognitive status and completed a short version of the learning paradigm for the purpose of stratification. Blinding, adverse effects and potential effects of tDCS on mood were systematically assessed. Figure 1a illustrates the design of the study.

### Participants

Participants were tDCS naïve, right-handed, healthy native English speakers (34 women, 16 men, mean ± SD years: 23.16 ± 3.79). Exclusion criteria followed standard tDCS safety criteria (e.g., history of seizures, metallic objects in the head, current depression or other psychiatric condition^25,26^). None of the participants reported use of recreational drugs or medication known to interact with tDCS effects (e.g., antidepressants, anxiolytics). A comprehensive neuropsychological test battery comprising paper-and-pencil as well as computerised tests (Cogstate test battery, https://cogstate.com/tests) was administered prior to the experimental procedures to ensure normal cognitive function in all participants (see Table 1 for details). The participants were stratified by age, sex and baseline learning ability on the short version of the learning paradigm (see below) and pairwise randomized to the stimulation groups. Both stimulation groups were comparable regarding demographic characteristics, baseline cognitive status and learning ability (see Table 1 for details). Written informed consent was obtained from each participant and the study was approved by the Human Research Ethics Committee of The University of Queensland. The study was conducted in strict accordance with the relevant ethical guidelines. The participants received AUD$50 upon study completion.

**Table 1:** Between Group Demographic and Neuropsychological Data.

	Sham HD-tDCS	Anodal HD-tDCS	Signif.
Age (yrs)	23.76 ± 4.33	22.56 ± 3.14	0.268
Education (yrs)	13.60 ± 1.73	13.92 ± 1.41	0.477
Mini-Mental State Examination (MMSE)^43^	29.68 ± 0.63	29.80 ± 0.50	0.458
**Verbal Fluency** (# correct/60 secs)			
Semantic Fluency	26.56 ± 5.85	24.96 ± 4.12	0.269
Phonemic Fluency	16.88 ± 5.80	16.20 ± 4.12	0.635
Boston Naming Test* (#correct)	14.16 ± 1.55	13.84 ± 1.46	0.456
**Colour-Word Interference (Stroop Test)** ^44^			
Colour Naming (secs)	24.80 ± 3.06	26.12 ± 3.28	0.148
Word Reading (secs)	18.96 ± 2.99	19.60 ± 2.31	0.401
Inhibition (secs)	39.20 ± 8.48	42.68 ± 6.57	0.111
**National Adult Reading Test (NART)** ^45^			
NART Error	19.16 ± 5.68	20.00 ± 4.50	0.565
NART IQ	112.16 ± 4.71	111.36 ± 3.73	0.508
**Hospital Anxiety and Depression Scale (HADS)** ^46^			
Depression	2.00 ± 2.33	2.32 ± 2.29	0.626
Anxiety	5.44 ± 2.74	5.44 ± 3.02	1.000
Cogstate Battery**			
Shopping List (# correct)	29.40 ± 2.42	28.80 ± 2.80	0.421
Identification (accuracy)	1.46 ± 0.14	1.51 ± 0.280	0.433
One Back (accuracy)	1.43 ± 0.13	1.41 ± 0.13	0.727
Two Back (accuracy)	1.36 ± 0.125	1.37 ± 0.14	0.868
Set-Shifting (# errors)	16.36 ± 7.21	14.28 ± 4.48	0.226
Paired Associative Learning (# errors)	28.00 ± 28.35	26.88 ± 26.96	0.887

Mean ± SD. Secs = Completion time in seconds.

*15-item short version^47^. **Cogstate test battery, https://cogstate.com/tests.

### Experimental learning paradigm

We employed an explicit, associative novel word learning paradigm. Participants were required to learn associations between black and white line drawings of Finnish farming equipment unknown to modern day users^8^, and an auditorily presented non-word “name”. The paradigm consisted of five training blocks and three interspersed recognition memory blocks. The latter were completed after the 1^st^, 3^rd^, and 5^th^ training blocks. During the training blocks, participants were instructed to actively memorize the names of each novel object. During the recognition blocks, participants were presented with the objects accompanied by a spoken non-word that was either the correct (previously paired) or incorrect (not previously paired) word and had to indicate whether each pairing was correct or not via a Yes/No response using the computer keyboard. This response mode (vs. overt naming) was chosen to facilitate implementation in future fMRI studies that will investigate the neural mechanisms underlying novel word learning improvement by HD-tDCS. Similar designs have been used in previous novel word learning studies^13,14,16^ and the task also bears significance for naturalistic language learning contexts (i.e., passive vocabulary).

### Training blocks

54 objects representing ancient Finnish farming equipment were used (see Fig. 1b for an example) during the training. During each of the five training blocks, all 54 pictures were presented once together with one of 54 pronounceable non-words (e.g., getolit). Non-words were 5–8 letters long (mean ± SD 5.96 ± 0.82) with vowel and consonant combinations that adhered to the rules of English and were selected from an online database^27^. Order of presentation was randomized across training blocks and four different randomizations were used across the group. Non-words bearing close resemblance to real English words were excluded. The chosen non-words were spoken by a female Australian native English speaker and digitally recorded at 44.1 kHz in a sound-proof room.

Each trial began with a fixation cross presented in the centre of the screen on a white background for 1500ms, followed by the object picture and a simultaneous auditory presentation of a non-word. Trials were separated by 500 ms. During each block, the object/name pairings were presented automatically on a computer screen with white background.

### Recognition blocks

During the recognition blocks, each tool object was presented in a pseudorandomised order on a white background with a simultaneous auditory presentation of a non-word that was either the “correct” or “incorrect” name for that object. Participants were instructed to use the computer keyboard to indicate whether the spoken non-word was the correct name for the object presented (left arrow = yes, right arrow = no). To assist participants with response selection, the words ‘yes’ and ‘no’ were presented at the bottom of the screen in their respective positions (left = yes, right = no). 27 objects were presented with “incorrect” non-word names that were previously presented with a different object during the training blocks. 27 objects were presented with their correct names. Number of correct responses (accuracy) and response latencies for correct responses were analyzed.

### Baseline learning ability

A short version of the learning paradigm (N = 12 different object/word pairings) assessed baseline learning ability prior to the training. This short version of the paradigm comprised three training blocks, each followed immediately by a recognition block. Learning success (total # correct responses) on this short version of the learning paradigm was used (with age and sex) to randomly assign participants to the stimulation groups (active vs. sham).

### High-Definition Transcranial direct current stimulation

tDCS was administered using a battery driven one-channel direct current stimulator (DC-Stimulator Plus, NeuroConn, Ilmenau, Germany). The HD-tDCS electrode montage comprised two concentric conductive rubber electrodes, a small round centre electrode (diameter = 2.5 cm), and a ring shaped return electrode (inner diameter = 7.5 cm; outer diameter = 9.8 cm^23^). The centre electrode was positioned over the temporo-parietal cortex as in previous studies that investigated novel word learning (i.e., position CP5 of the 10–20 EEG system^28^). We have recently provided evidence that this set-up (a) induces regionally and task-specific stimulation effects^23,24^ and (b) allows for focal current delivery (for details of current distribution, intensity and penetration see^29^). Stimulation of this region with conventional tDCS improved both implicit and explicit new word learning in healthy young individuals^12–15^. The current was ramped up immediately prior to the first training block over 10 seconds to 1 mA during both stimulation conditions. Afterwards, it remained constant for 20 min (anodal tDCS) or 40 seconds (sham tDCS) before ramping down (over 10 sec). This protocol has been shown to induce effective blinding of participants in the sham tDCS group by inducing a similar physical sensation as active stimulation^22^. Investigator blinding was achieved by the “study mode” of the DC stimulator (i.e., a predefined code triggered active or sham tDCS). Codes were assigned by a researcher not involved in conducting the experiments.

### Mood, adverse effects and blinding

Mood was assessed before and after each daily learning session using the Visual Analogue Mood Scale (VAMS^30^). The VAMS assessed current positive and negative emotional states on visual analogue scales ranging from 0 to 100 (i.e., afraid, confused, sad, angry, energetic, tired, happy, tense). Higher scores indicate greater intensity. Adverse effects were assessed using a self-report questionnaire developed by Brunoni *et al*.^31^. The participants rated the presence and intensity of a range of possible adverse events (1 = absent, 2 = mild, 3 = moderate, 4 = severe, see Table 2). Participant blinding was assessed at the completion of training. Participants were asked the following: “What type of stimulation do you believe you received? (a) real stimulation (anodal tDCS), (b) placebo, or fake stimulation (sham tDCS), or (c) unsure”?

**Table 2:** Mean (±SD) Adverse Effects Ratings.

Symptom	Sham	Anodal	Between group comparison
Headache	1.12 ± 0.33	1.12 ± 0.44	p = 1.000
Neck pain	1.24 ± 0.52	1.12 ± 0.44	p = 0.384
Scalp pain	1.32 ± 0.56	1.12 ± 0.44	p = 0.165
Tingling	1.88 ± 0.67	2.16 ± 0.47	p = 0.093
Itching	1.48 ± 0.71	1.80 ± 0.71	p = 0.118
Burning	1.52 ± 0.77	1.56 ± 0.71	p = 0.850
Redness	1.04 ± 0.20	1.00 ± 0.00	p = 0.322
Sleepiness	2.40 ± 0.76	2.24 ± 0.72	p = 0.451
Concentration	1.72 ± 0.11	1.72 ± 0.68	p = 1.000
Mood Change	1.16 ± 0.37	1.08 ± 0.28	p = 0.394

### Statistical analysis

All analyses were conducted on the full sample (sham/anodal, n = 25/25). Potential differences between the stimulation groups in baseline cognitive status and adverse effects were assessed using independent samples t-tests (2-tailed). Mood ratings were analysed by combining scores into two composite measures: positive affect and negative affect following our previous studies^23,32,33^. Change scores for positive and negative affect (post-pre) were calculated and compared between groups using independent samples t-tests (2-tailed). Chi-square tests assessed potential group differences in blinding success.

Effects of active-tDCS on recognition accuracy (# correctly identified object names) and response latencies for correct responses over the three recognition blocks were analysed using a 2 × 3 mixed-model analysis of variance (ANOVA) with the three time-points for the recognition blocks (TIME) as a within-subjects variable and stimulation group (STIMGROUP) as a between-subjects variable. A separate model assessed potential baseline differences in learning ability between the two groups. Data was found to be normally distributed when assessed with the Shapiro-Wilk test. For ANOVA, all data met the assumption of homogeneity of variance using Levene’s test. The Greenhouse-Geisser correction was applied when a violation to the assumption of sphericity was identified using Mauchly’s test. The datasets generated during the current study are available from the corresponding author on reasonable request.

## Results

### Adverse effect, mood, and blinding

Only 36% (n = 18) of the participants correctly guessed which type of stimulation they had received (Incorrect: 34%, n = 17; Unsure: 30%, n = 15). The stimulation groups did not differ in this regard (χ^2^(2) = 0.125, p = 0.939; Sham/Anodal, Correct: 9/9, Incorrect: 8/9, Unsure: 8/7). Therefore, blinding was successful. All participants tolerated the stimulation well and only mild adverse effects were reported, with no differences between the stimulation groups (all p > 0.09; See Table 2). Group differences in mood change measured before and after stimulation and training did not reach significance (positive affect: t(48) = −1.876, p = 0.067, Mean ± SD, Sham: −13.58 ± 21.12, Anodal: −3.34 ± 17.28; negative affect: t(48) = −0.462, p = 0.646, Mean ± SD, Sham: 0.57 ± 5.90, Anodal: 1.30 ± 5.31).

### Baseline learning ability

Both accuracy and response latencies were comparable in the two stimulation groups. Specifically, there were no overall differences between the stimulation groups in learning success (Accuracy: STIMGROUP, *F*(1, 48) = 0.121, p = 0.730) and the degree of learning across the three recognition trials (Accuracy: TIME × STIMGROUP, *F*(1.59, 76.32) = 0.310, p = 0.684). Response latencies were also comparable in the two groups (STIMGROUP, *F*(1, 48) = 0.141, p = 0.709; TIME × STIMGROUP, *F*(1.42, 68.16) = 1.6, p = 0.213, Fig. 1).

## Online Learning

### Overall learning

**Figure 2:**
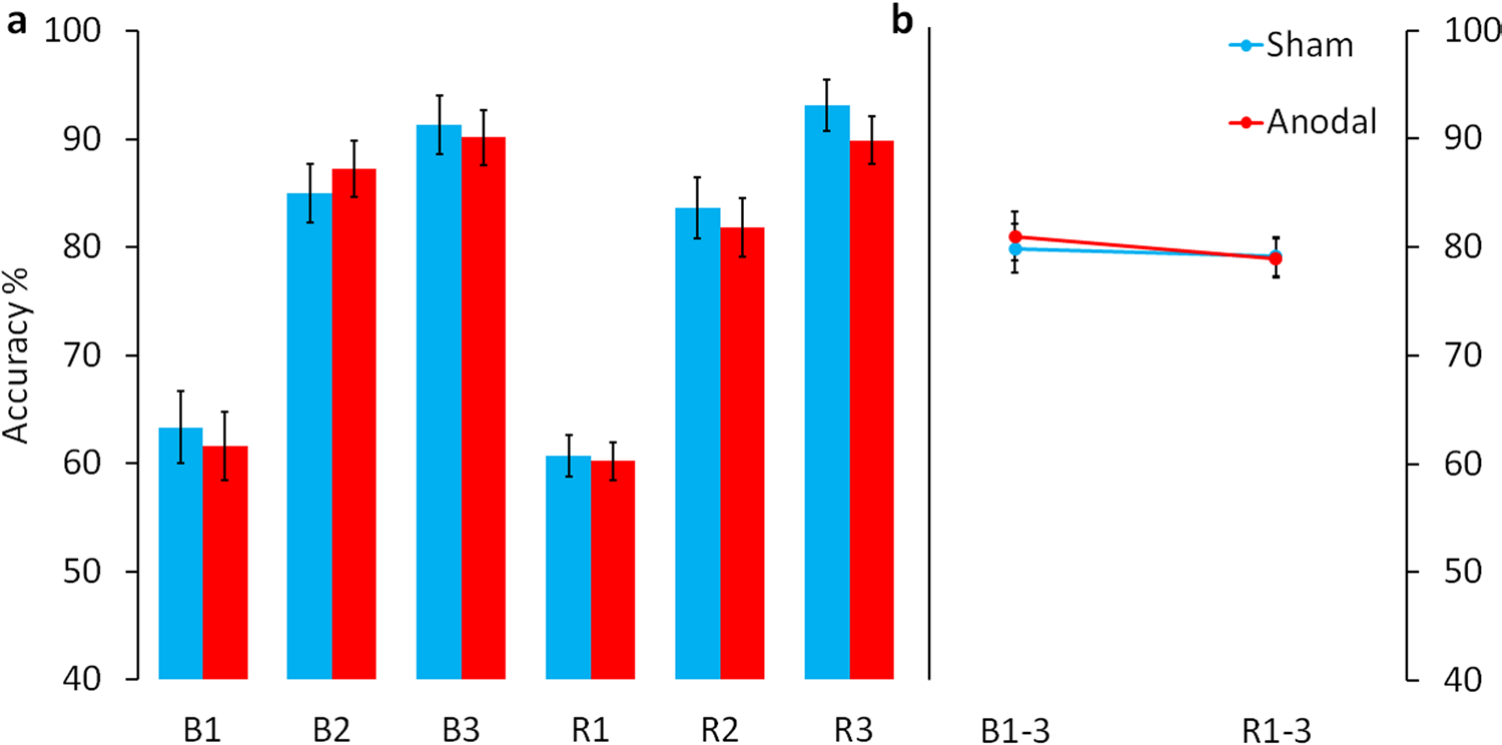
HD-tDCS effects on response accuracy. **(a)** Shows mean accuracy (% correct responses) for sham and anodal HD-tDCS groups during the three baseline learning recognition blocks (B1–3) and the three recognition blocks during the experimental phase (R1–3). All participants successfully learning the novel vocabulary (>90% correct at R3). **(b)** Illustrates the main effect of stimulation averaged across the recognition blocks for baseline learning (B1–3) and the experimental phase (R1–3). Shows mean accuracy (% correct responses) for sham and anodal HD-tDCS averaged across the recognition blocks for baseline learning (B1–3) and the experimental phase (R1–3). Sham and anodal HD-tDCS subjects showed comparable accuracy at baseline and during the experimental phase. Error bars indicate standard error of the mean (SEM).

**Figure 3:**
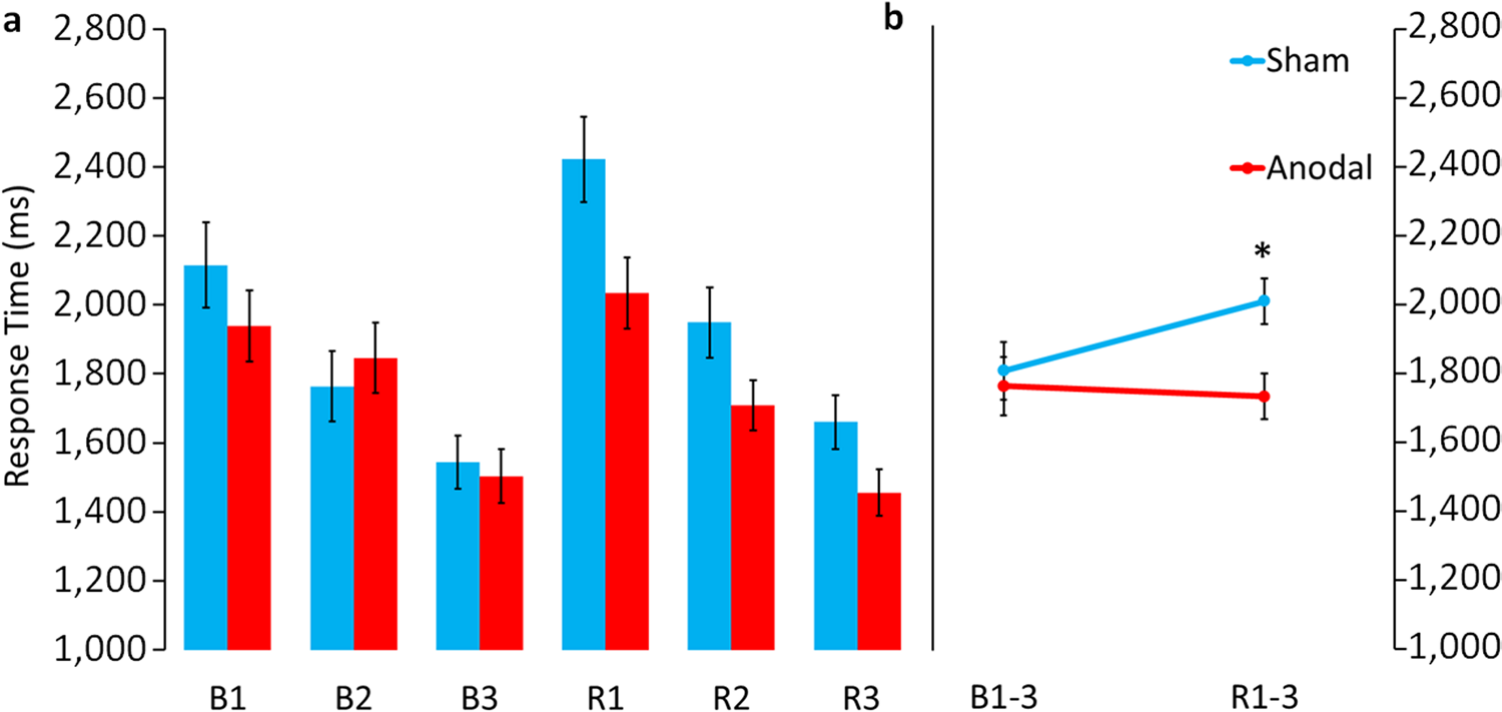
HD-tDCS effects on response latencies. **(a)** Shows mean response latencies for with correctly identified pairings for sham and anodal HD-tDCS groups during the three baseline learning recognition blocks (B1–3) and the three recognition blocks during the experimental phase (R1–3). **(b)** Illustrates the main effect of stimulation averaged across the recognition blocks for baseline learning (B1–3) and the experimental phase (R1–3). Sham and anodal HD-tDCS subjects were comparable at baseline. Immediate effects of HD-tDCS at R1 were maintained at R2 and R3. Error bars indicate standard error of the mean (SEM). *Indicates significant main effect of stimulation during experimental phase (p = 0.005).

Participants in both groups successfully learned associations between the novel objects and their non-word names. This was evidenced by a significant increase in accuracy scores (TIME, *F*(1.30, 62.50) = 202.287, p < 0.001) and faster response times (TIME, *F*(1.34, 64.15) = 63.602, p < 0.001) over the three recognition blocks (Figs 2 and 3).

### Accuracy

There was no difference between stimulations groups in overall accuracy scores (STIMGROUP, *F*(1, 48) = 0.003, p = 0.953; Mean ± SEM, Sham: 42.73 ± 0.96, Anodal: 42.64 ± 0.96). Both groups showed near identical learning curves and the degree of learning across the three recognition blocks was not different (Accuracy: TIME × STIMGROUP, *F*(1.3, 62.50) = 0.082, p = 0.840, Fig. 2).

### Response latencies

Active HD-tDCS resulted in overall faster response times over the three recognition blocks (STIMGROUP, *F*(1, 48) = 8.806, p = 0.005; Mean ± SEM, Sham: 2010.50 ± 66.20, Anodal: 1732.69 ± 66.20). Active HD-tDCS resulted in immediate improvement from the first recognition block on, which was maintained across blocks as indicated by the non-significant interaction of TIME × STIMGROUP (*F*(1.34, 64.15) = 1.316, p = 0.267, Fig. 3).

## Discussion

This study investigated for the first time whether focal HD-tDCS of the left temporo-parietal cortex can improve novel word learning ability. In a between-group design, two carefully matched groups of healthy young individuals received either active or sham HD-tDCS. In the absence of differences in demographic and cognitive status and baseline learning ability on a task with the same structure and similar stimuli, active HD-tDCS improved performance during the experimental phase compared to sham HD-tDCS. Specifically, while accuracy was unaffected by the stimulation, HD-tDCS resulted in faster retrieval of correct word-picture associations during the recognition phases. No group differences were found regarding potential mood changes after the end of the stimulation and blinding was equally effective in both groups. Therefore, our positive results cannot be explained by these variables. We will discuss these results in more detail below.

Only a handful of studies had previously explored potential tDCS effects on novel word learning using conventional tDCS montages^12–16^. While the experimental procedures and exact stimulation parameters varied substantially, all studies reported beneficial effects of left temporo-parietal cortex stimulation on novel word learning. Of note, Fiori *et al*.^16^ replicated beneficial effects in a group of elderly participants using exactly the same design as in a previous study of the same group^13^, but failed to show positive stimulation effects in younger adults. However, the relatively simple task that was used in this study (learning of associations between 20 picture and non-word pairings) may have prevented further gains by tDCS and the young group performed close to ceiling levels. Similarly, only studies that involved challenging tasks (e.g., by using large stimulus sets, implicit learning paradigms, serial recall or translation tasks) reported increased accuracy rates due to tDCS^12,14,15^, while easier tasks involving fewer stimuli^13,16^ or easier forced choice decisions between stimuli like the present study mainly resulted in reduced latency of correct responses. Therefore, our results are consistent with previous studies and provide strong evidence that temporo-parietal cortex tDCS can enhance novel word learning in healthy individuals.

However, all previous studies of novel word learning employed conventional tDCS montages, which prevents attributing positive stimulation effects to the target region due to current spread to regions located in between the active and reference electrodes. Moreover, previous studies that combined conventional tDCS with functional brain imaging demonstrated not only local changes in activity at the stimulation site, but also activity modulations in remote regions and networks^32,34,35^. Therefore, it is likely that functionally connected fronto-temporal language regions^1,8^ or domain general regions supporting attention and executive control that are important during early stages of vocabulary acquisition^9^, were affected by tDCS. In addition, the use of small reference electrodes can result in inhibition of the right prefrontal cortex. This may have contributed to improved learning ability, particularly in studies that recruited elderly participants^13,16^, because hyperactivity of the right prefrontal cortex has been linked to impaired language processing in advanced age^32,33,36^.

In contrast, the HD-tDCS set-up that was used in the present study allows for more focal current delivery, as suggested by recent computer simulations of tDCS effects^18,21,29^ and also a recent functional near infrared spectroscopy (fNIRS^37^). Therefore, the positive effects of HD-tDCS in the present study are unlikely explained by current spread to distant brain regions and our group has recently demonstrated region- and task-specific stimulation effects using the same set-up that was used in the present study^23,24^. Importantly, temporo-parietal HD-tDCS does not result in current spread to primary or pre-motor cortices^29^. While this makes it unlikely that our results were mediated by facilitation of motor responses, it would have been beneficial to include a control task that assesses potential effects of temporo-parietal HD-tDCS on motor speed.

It also needs to be acknowledged that our study was not designed to test the regional specificity of the stimulation effects. Regionally specific effects of HD-tDCS have recently been demonstrated by our group^23^. In this study, HD-tDCS of both the left and right dorsolateral prefrontal cortex improved conflict adaptation during a visual flanker task. In contrast, HD-tDCS of two control sites (i.e., left and right primary motor cortex) affected neither (a) latency for responses with the hand contralateral to the respective stimulation sites nor (b) conflict adaptation. However, only one of the previous studies that employed conventional temporo-parietal tDCS on novel word learning employed a control site^13^. This study demonstrated more pronounced learning during temporo-parietal stimulation compared to both sham and active right-sided temporo-occipital tDCS, but did not address the specific neural processes that were affected by the stimulation. However, the neurocognitive processes underlying novel word learning have been investigated in numerous behavioral and imaging studies (for review see^1^). These studies have suggested that the left temporo-parietal cortex is part of a larger “dorsal-audio-motor interface” implicated with the acquisition of new vocabulary by generating sensory representations of novel phonological forms and by a rehearsal process in phonological working memory. For example, there is evidence from functional imaging that the temporo-parietal cortex is activated during phonological encoding and more pronounced activity in this region during subsequent retrieval is positively correlated with memory performance for novel word forms^38^. In line with these findings, Takashima *et al*.^39^ have demonstrated that the temporo-parietal cortex is involved in establishing long-term holistic representations of novel words. A recent tDCS study further highlighted the importance of this region in establishing novel word forms in long-term memory^15^. These authors were able to demonstrate that conventional tDCS of the left temporo-parietal cortex enhanced phonological stability of newly learned whole word forms indicated by reduced phoneme migrations during a serial word recall task.

While these studies provided evidence for a specific role of the temporo-parietal cortex in establishing novel word forms, it is important to note, that both conventional tDCS^33,40^ and also more targeted stimulation of circumscribed brain regions by transcranial magnetic stimulation^41,42^ have been shown to affect functionally connected brain regions in imaging studies. Therefore, similar non-localized effects are to be expected for more focal tDCS protocols and future studies are necessary to establish the exact neural locus underlying improved novel word learning due to HD-tDCS. While the design of the present study was not developed to establish the contribution of specific sub processes contributing to enhanced novel word learning, the short block duration with interspersed recognition trial blocks allows portability of the paradigm into an MRI environment. Moreover, our group has recently demonstrated that the HD-tDCS montage used in the present study is safe for use during functional magnetic resonance imaging (fMRI). This will allow future studies to determine the neural mechanisms underlying effective behavioral modulation by HD-tDCS.

In sum, our results demonstrate that HD-tDCS of the temporo-parietal cortex can enhance access to newly learned words in healthy young individuals. Future studies that combine functional imaging with this novel approach will contribute to establishing the exact locus of (HD-) tDCS action in the human brain.
